# Advances in epigenetic modifications of autophagic process in pulmonary hypertension

**DOI:** 10.3389/fimmu.2023.1206406

**Published:** 2023-06-16

**Authors:** Min Mao, Shasha Song, Xin Li, Jiayao Lu, Jie Li, Weifang Zhao, Hanmin Liu, Jingxin Liu, Bin Zeng

**Affiliations:** ^1^ Department of Pediatric Pulmonology and Immunology, West China Second University Hospital, Sichuan University, Chengdu, China; ^2^ Key Laboratory of Birth Defects and Related Diseases of Women and Children (Sichuan University), Ministry of Education, Chengdu, China; ^3^ National Health Commission (NHC) Key Laboratory of Chronobiology (Sichuan University), Chengdu, China; ^4^ The Joint Laboratory for Lung Development and Related Diseases of West China Second University Hospital, Sichuan University and School of Life Sciences of Fudan University, West China Institute of Women and Children’s Health, West China Second University Hospital, Sichuan University, Chengdu, China; ^5^ Sichuan Birth Defects Clinical Research Center, West China Second University Hospital, Sichuan University, Chengdu, China; ^6^ College of Pharmacy, Shenzhen Technology University, Shenzhen, China; ^7^ Marketing Department, Shenzhen Reyson Biotechnology Co., Ltd, Shenzhen, China; ^8^ Nanjing Evertop Electronics Ltd., Nanjing, China; ^9^ Quality Management Department International Registration, North China Pharmaceutical Co., Ltd. (NCPC), Hebei Huamin Pharmaceutical Co., Ltd., Shijiazhuang, China

**Keywords:** pulmonary hypertension, autophagy, epigenetic modifications, histone modifications, DNA methylation, alternative RNA splicing

## Abstract

Pulmonary hypertension is characterized by pulmonary arterial remodeling that results in increased pulmonary vascular resistance, right ventricular failure, and premature death. It is a threat to public health globally. Autophagy, as a highly conserved self-digestion process, plays crucial roles with autophagy-related (ATG) proteins in various diseases. The components of autophagy in the cytoplasm have been studied for decades and multiple studies have provided evidence of the importance of autophagic dysfunction in pulmonary hypertension. The status of autophagy plays a dynamic suppressive or promotive role in different contexts and stages of pulmonary hypertension development. Although the components of autophagy have been well studied, the molecular basis for the epigenetic regulation of autophagy is less understood and has drawn increasing attention in recent years. Epigenetic mechanisms include histone modifications, chromatin modifications, DNA methylation, RNA alternative splicing, and non-coding RNAs, which control gene activity and the development of an organism. In this review, we summarize the current research progress on epigenetic modifications in the autophagic process, which have the potential to be crucial and powerful therapeutic targets against the autophagic process in pulmonary hypertension development.

## 1 Introduction

Autophagy has been implicated in multiple physiological processes that are important for human health and disease (1, 2). It is an adaptive process that occurs in response to different forms of stress, including hypoxia, infection, and nutrition deprivation. The damaged organelles, pathogen, aggregated proteins, or long-lived proteins are collected, delivered to lysosomes, digested by lysosomal hydrolases, and recycled to produce amino acids and fatty acids necessary for ATP production and cellular response (3). Multiple signaling pathways and autophagy-related proteins (ATG proteins) have been implicated in completing the autophagic process. So far, according to the human autophagy database developed by the Laboratory of Experimental Cancer Research headed by Dr. Guy Berchem, more than 200 different autophagic genes directly or indirectly modulating the autophagic process have been discovered. More than 40 genes encoding ATG proteins have been identified in yeast, and most of the genes (ATG1-ATG10, ATG12-ATG14, ATG16-ATG18) are conserved between yeast and mammals, which indicates the autophagic process as an intracellular evolutionarily conserved degradation process (4). The so-called core ATG proteins essential for autophagic process completion undergo different modifications, including epigenetic modifications, acetylation, phosphorylation, and ubiquitylation, which affect the role of autophagy-related proteins in the autophagic process. Numerous signaling pathways serve as upstream regulators of autophagy, including the NF-κB, STAT3, p53, FOXO, Sirt1, and HDAC signaling pathways. These signaling pathways also have significant influences on angiogenesis, endothelial-to-mesenchymal transition, and programed cell death, resulting in vascular remodeling and vascular resistance. Epigenetics refers to the regulation of epigenomic gene expression by epigenetic alterations (DNA methylation, histone modifications, and alternative RNA splicing) that are independent of changes in gene sequences and are heritable. Factors such as DNA methylation, histone modifications, and alternative RNA spliced isoforms are responses to changes in environmental stimuli that interact to regulate gene expression and control cellular phenotypes, all of which are necessary to maintain environmental stability in the body and contribute to normal physiological functioning. The epigenetic modifications of these signaling pathways affect the expression, stability, and function of autophagy-related proteins.

**Figure 1:**
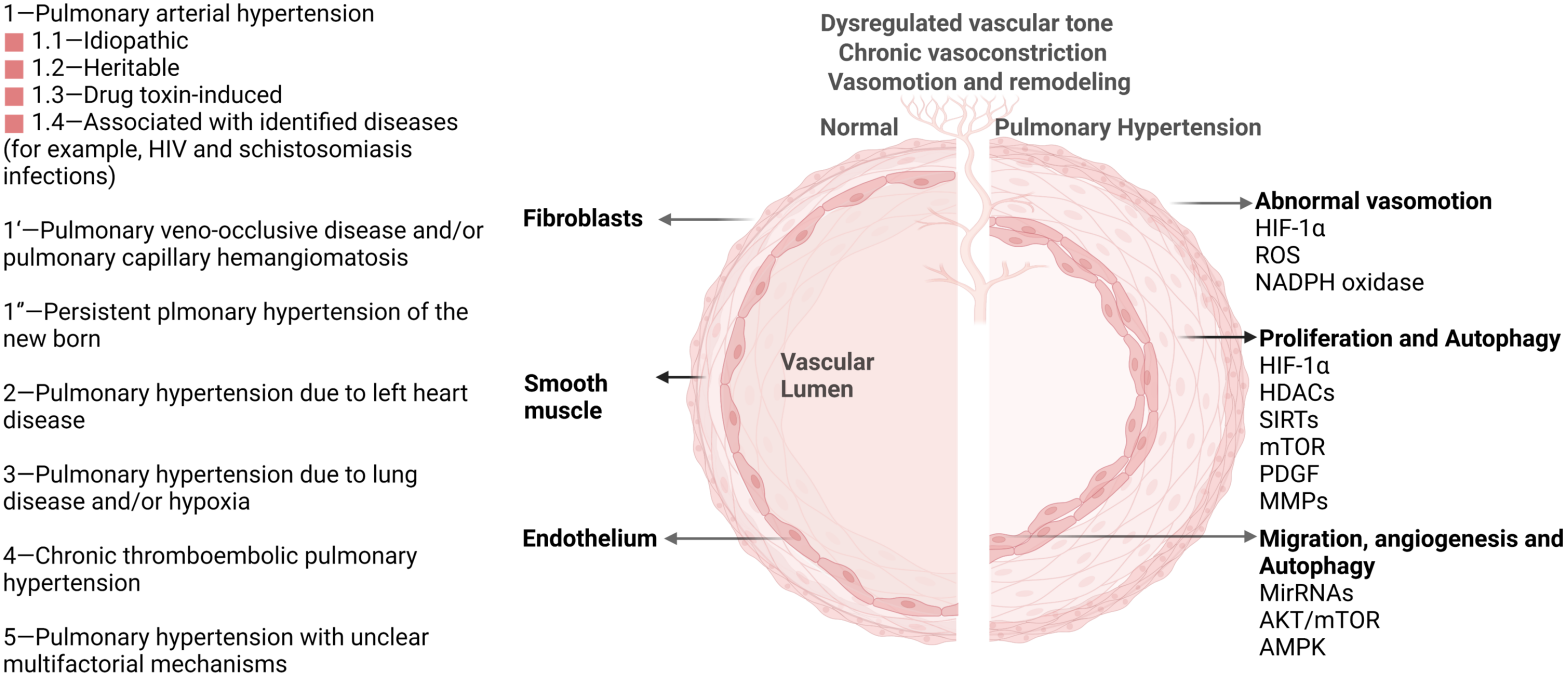
Pulmonary hypertension classification and vascular pathological progression in pulmonary hypertension. As agreed at the 6th World Symposium on Pulmonary Hypertension in 2018, pulmonary hypertension is now classified into five groups (Left) (6). Multiple signaling pathways (HIF-1α, HDACs, AKT/mTOR, et. al) participate in the pathological progression and phenotype in PH (Right). These important pathways also activate autophagy and impact PH progression through the autophagy process.

Direct links exist between autophagy and pulmonary hypertension (PH) according to various research. Pulmonary hypertension is a fatal and heterogeneous disease characterized by elevated pulmonary vascular resistance and pulmonary artery pressure, resulting in the remodeling of the pulmonary vasculature (5). PH is divided into five categories according to inherited or unknown causes, heart disease, lung disease, the blockage of blood vessels, or other medical conditions such as some blood disorders (**Figure 1**) (6). The pathologic process of PH begins with vasoconstriction and remodeling of the small pulmonary arteries, leading to an increase in pulmonary vascular resistance (PVR). This can be caused by a variety of factors including genetic mutations, viral infections, and exposure to toxins such as cigarette smoke. As PVR increases, the right ventricle must work harder to pump blood through the lungs into the left side of the heart, leading to right ventricular hypertrophy and eventually right heart failure (7). The primary cellular mechanism underlying the pathologic progression of PH is abnormal smooth muscle cell and endothelial cell proliferation. As the disease progresses, these cells proliferate excessively and invade the surrounding extracellular matrix, leading to a narrowing of the lumen of the pulmonary artery and increasing the resistance to blood flow. Additionally, there is also a shift towards a pro-inflammatory and thrombotic state, further contributing to disease progression. The histopathological changes in PH include intimal fibrosis, medial hypertrophy, and adventitial remodeling. Intimal fibrosis refers to the thickening of the intima, or innermost layer, of the pulmonary artery due to a buildup of collagen and other extracellular matrix proteins. Medial hypertrophy refers to the thickening of the media, or middle layer, of the pulmonary artery due to an increase in smooth muscle cell mass. Adventitial remodeling involves changes in the outermost layer of the pulmonary artery, including inflammation and fibrosis (8). Autophagy has been discovered to be upregulated in PH patients compared with healthy controls. Administration of an autophagy antagonist prevents the progression of experimental pulmonary hypertension (9). This phenomenon provides a clue about the importance of discovering the underlying key regulatory mechanism of autophagy in PH. As a homeostatic mechanism essential for cell survival, autophagy activation under stress affects various pathological processes of pulmonary hypertension, including disrupted redox balance, blocked apoptosis, inflammation, angiogenesis, vascular calcification, and remodeling (10, 11). Understanding these mechanisms of autophagy will provide novel therapeutic targets for PH. Despite the great advances in the investigation of the autophagic process in PH, the mechanism of autophagy remains elusive. In this review, we focus on epigenetic modification in autophagic process of PH.

## 2 The autophagic process in PH

**Figure 2:**
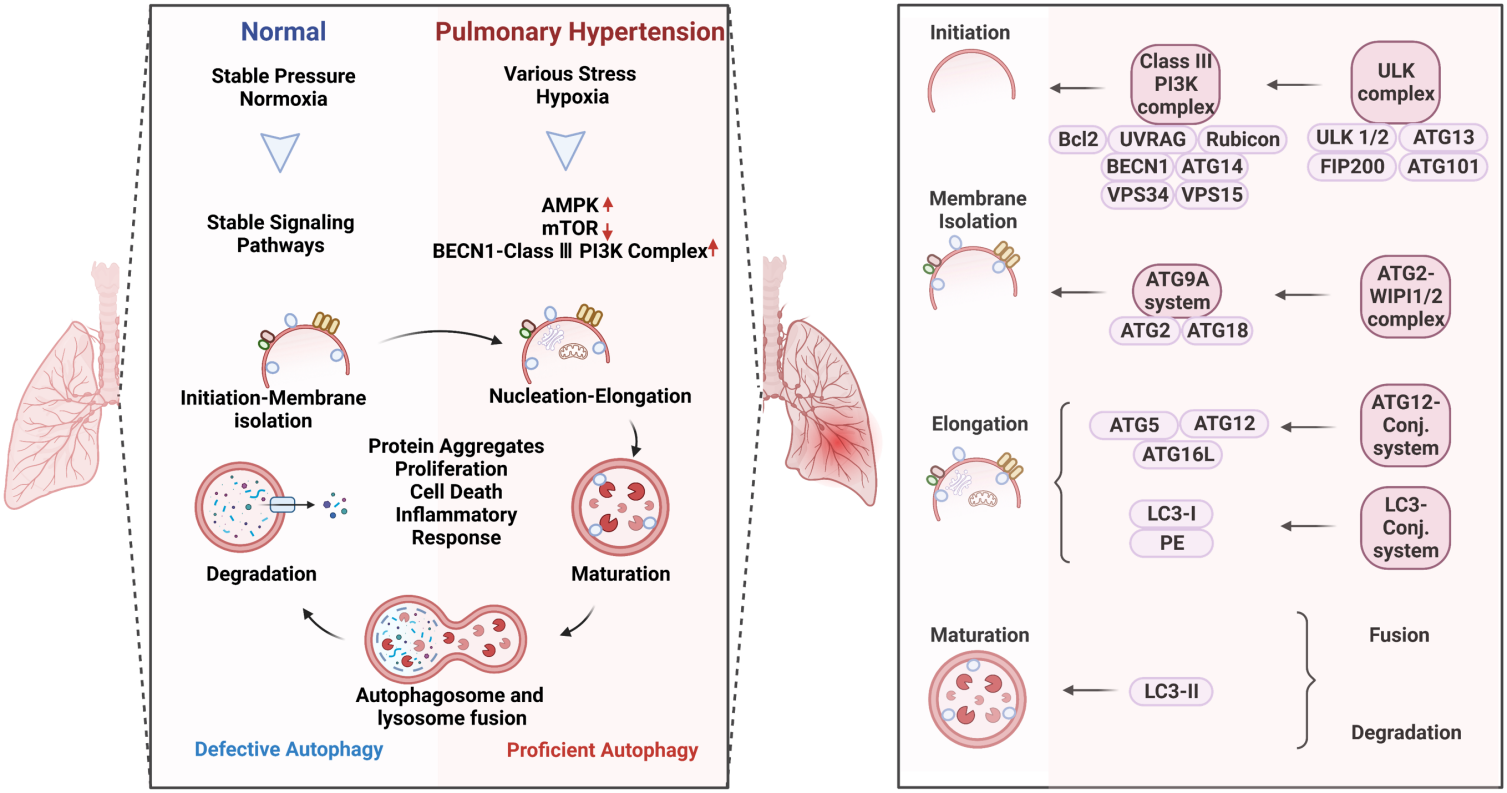
Autophagy activation in pulmonary hypertension (Left) and the specific role of the complexes in the process of autophagy (Right). Autophagy is a process of intracellular waste degradation and reuse in response to different microenvironment stresses. Its activation promotes the survival and proliferation of pulmonary vascular endothelial cells while also regulating different cell types such as alveolar macrophages, smooth muscle cells, and fibroblasts, thereby participating in the occurrence and development of pulmonary hypertension. In addition, autophagy can also promote cellular metabolic balance, reduce oxidative stress, and inflammatory response, and is expected to become a new target for the treatment of pulmonary hypertension.

Autophagy is a conserved adaptive process that has played an important role in human disease. During the development of PH, the cellular autophagy mechanism, which removes useless materials such as aging, damaged, and abnormal cells, plays different roles at different stages of progression. In the early stage of PH, autophagy plays a protective role. Studies have shown that the expression levels of autophagy-related proteins are low in the lung tissues of patients with PH, but an increase in autophagy levels through drug or genetic intervention can alleviate the pathological damage of PH (12). Autophagy can clear harmful molecules such as excess proteins, organelles, and oxidative products that cause cell death, promote cell survival, and function maintenance. In addition, in the early stage of PH, autophagy can also regulate the reactive oxygen species (ROS) level of endothelial cells, inhibit pulmonary vascular contraction, and maintain normal blood flow. In the mid-to-late stages of PH, autophagy plays a dual role. On the one hand, autophagy activation can protect pulmonary artery endothelial cells (PAECs) from apoptosis (13). Endothelial dysfunction and apoptosis are key features of PH, and autophagy activation can maintain endothelial cell survival by clearing damaged organelles and proteins. On the other hand, excessive autophagy activation in the mid-to-late stages of PH can lead to abnormal cellular metabolism and inflammation, which may contribute to the progression of PH (14, 15). Studies have shown that the levels of autophagy-related proteins increase significantly in the lungs of patients with PH, suggesting a potential link between autophagy dysregulation and disease progression. Therefore, it is important to strike a balance between autophagy activation and inhibition to achieve optimal therapeutic effects in PH (**Figure 2**, left).

Autophagy refers to macroautophagy, mitophagy, and chaperone-mediated autophagy (**Figure 2**, right). The autophagic process involves five core complexes: (i) the ULK kinase complex, consisting of ULK1/2, ATG13, RB1CC1/FIP200, and ATG101; (ii) the ATG9A/ATG2-WIPI1/2 trafficking system; (iii) the class III PI3K complex, which includes VPS34, Beclin 1, p115, and either ATG14 in PI3KC3 complex I or UVRAG in complex II; (iv) WIPI proteins and their interaction partner ATG2; and (v) two ubiquitin-like proteins and their conjugation machinery. Autophagy acts as a double-edged sword in human diseases which can promote cell survival or apoptosis under different stresses (16). These opposite effects could possibly be attributed to the different modifications of ATG-related proteins in a context-dependent manner. In response to different pathological stimulus, autophagy is abnormally activated in PH patients (9). The research surrounding the altered autophagy phenotypes has bloomed since work on autophagy won the Nobel Prize in 2016, making great progress in the understanding of the underlying mechanisms in PH.

The key autophagy-related genes (LC3B-II, Beclin1) and upper signaling molecules (AMPK, mTOR, ·BMP, ROS, NF-κB) have been discovered activated and playing important roles in regulating autophagy in PH. Activated autophagy promotes the proliferation of PASMC (pulmonary arterial smooth muscle cells) and PAEC (pulmonary arterial endothelial cells), inhibits ROS production, and regulates mitochondrial function in PH (11). Autophagy plays a dual role in endothelial cells. On the one hand, it promotes the survival and adaptation of these cells under stress conditions by removing damaged organelles and recycling nutrients to provide energy for the cell. On the other hand, excessive autophagy can result in endothelial dysfunction and apoptosis, leading to impaired vascular homeostasis and contributing to PH progression (13). The underlying mechanism involves modulation of key signaling pathways such as AKT/mTOR, AMPK, and ERK1/2. In smooth muscle cells, autophagy has been shown to regulate the proliferation and migration of these cells (14). Specifically, inhibition of autophagy leads to reduced smooth muscle cell proliferation and increased apoptosis. This effect is mediated through the modulation of RhoA and mTOR pathways. In addition, autophagy also plays a role in the regulation of immune cells during PH development. Autophagy promotes antigen presentation by dendritic cells, enhances T-cell survival, and regulates macrophage polarization towards an anti-inflammatory phenotype (17). Dysregulation of autophagy in immune cells can lead to inflammation, promoting progression of PH. The exact molecular mechanisms behind autophagy-mediated immune cell regulation in PH are still being investigated. Overall, autophagy is a complex process that plays a multifaceted role in PH pathogenesis, affecting multiple cell types and signaling pathways. Increasing our understanding of the specific molecular mechanisms underlying autophagy in PH may enable development of new therapies targeting this process to improve patient outcomes.

Multiple signaling genes are important in human diseases through affecting various downstream pathways. Various modifications play key roles in regulating the expression or functions of the signaling molecules themselves or downstream molecules. These modifications include post-transcriptional, post-translation, and pre-transcriptional modifications. Epigenetics refers to histone modification and chromatin remodeling, which means reversible and hereditary changes in gene expression without alterations in DNA sequences. Post-translational modification of histones, including acetylation, methylation, phosphorylation, SUMOylation, ubiquitination, and ADP-ribosylation, occur mainly in the N-terminal tails and have profound effects on chromatin structure. Epigenetic modification affects autophagosome formation, autophagy-related protein expression, and signaling pathway activation and potentially functions as the PTM switch to regulate autophagy (18). As histone acetylation or methylation modifying enzymes, HDACs, SIRT1, SIRT3, and BRD4 proteins are confirmed with significant alternation in expression levels and play important roles in the proliferation, inflammatory and fibrotic phenotypes of vascular cells (19). Additionally, these signal molecular genes regulating autophagy are also reported to be impacted by epigenetic modifications such as acetylation (Ac) and methylation (Me), which affect the chromatin state, thus altering expression of specific genes (20, 21).

## 3 Acetylation signaling of autophagy relevant to pulmonary hypertension

**Figure 3:**
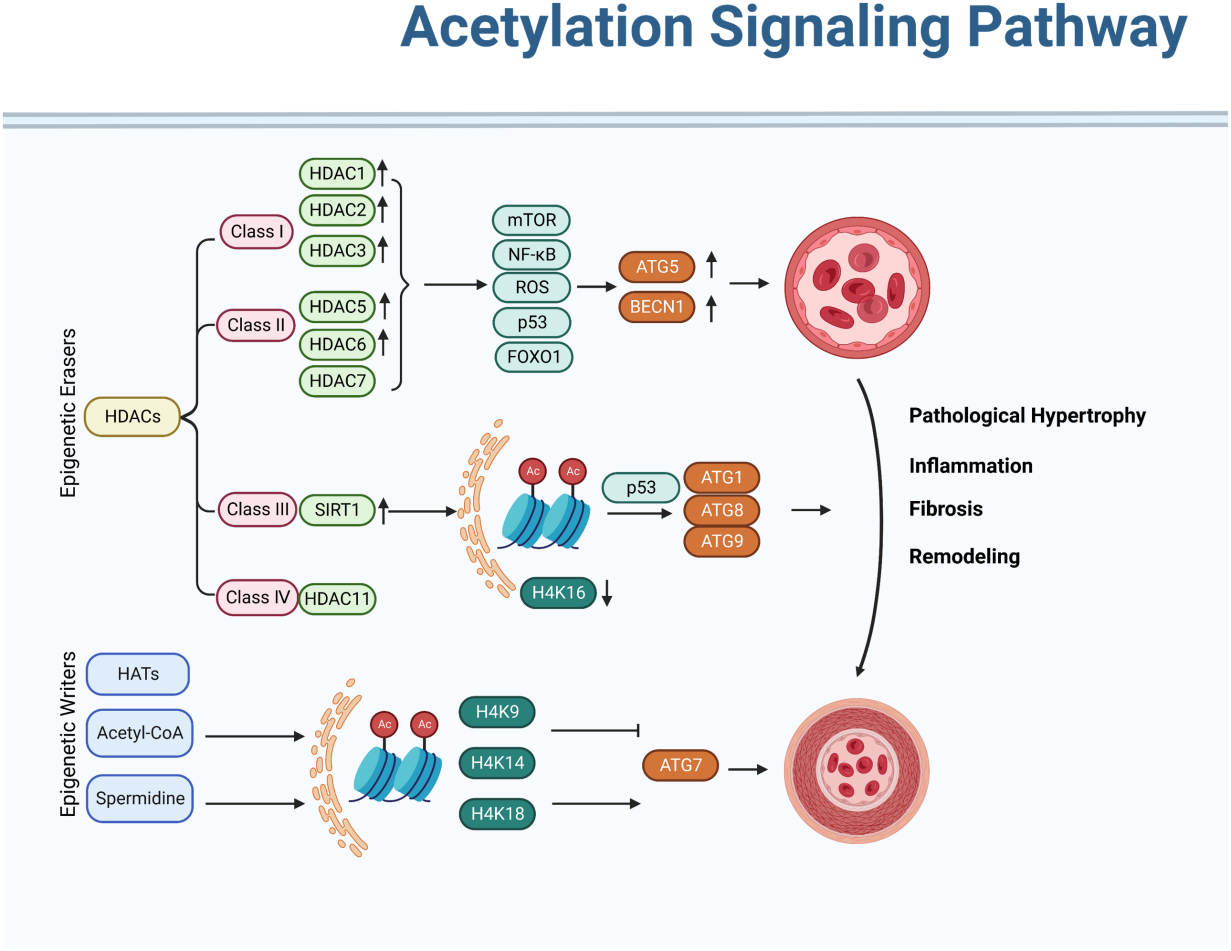
Acetylation signaling pathway in regulating autophagy activation of pulmonary hypertension. Acetylation modifications can modulate the activity of autophagy, thereby exerting a protective effect against pulmonary hypertension. In addition, various drugs can also be used to treat pulmonary hypertension by modifying acetylation modifications. However, further research is needed on the specific mechanism and effect of acetylation modification in pulmonary hypertension.

Acetylation refers to the process of transferring and adding acetyl groups to protein lysine residues or protein N-terminus under the catalysis of acetyltransferases (or non-enzymes). Histone acetylation affects the pathological progression of PH through repressing ATG genes transcriptionally. Research has shown that the acetylation of autophagy-related proteins plays an important role in the regulation of autophagy in PH. Specifically, increased levels of acetylated proteins such as LC3B and ATG7 have been observed in animal models of PH and in human patients with the disease. These proteins are key components of the autophagy machinery and their acetylation has been shown to impair autophagic function (22). We summarize recent research which has shown that histone-modifying enzymes occupy an important position in regulating autophagic process of PH (**Figure 3**).

Histone lysine acetyltransferases (HATs) and deacetylases (HDACs) gene families are the main regulators of histone acetylation (19). Acetylation of histone tails neutralizes positively charged lysine, which is thought to disrupt the interaction between the tail and negatively charged nucleosome DNA, thereby promoting the opening of chromatin and thus positive transcription. At present, 18 HDACs have been found in humans belonging to four categories (I, II, III, and IV). Among them, 11 subtypes, such as class I, II, and IV, were Zn2+-dependent proteins. Seven subtypes of the class III subgroup, Sirt1~7, use NAD+ as the catalytic active site. Acetylation of lysine at histone tails is highly dynamic and important for the regulation of chromatin structure, transcription, and DNA repair. Acetylation of non-histones, including tumor suppressors and oncogenes (i.e., p53, Rb, and Myc), regulates protein stability, DNA binding, protein interactions, enzyme activity, or protein localization.

HDAC1, HDAC2, and HDAC3 are elevated in PH fibro6blasts (23). Additionally, HDAC1 and HDAC5 have also been reported as being activated in human idiopathic PAH (IPAH) lung homogenates (24). HDACs play crucial roles in the control of pathological hypertrophy, inflammation, fibrosis, restenosis, and left ventricular (LV) cardiac remodeling in various preclinical models of LV failure (25). The prototypical HDAC inhibitor (HDACi), trichostatin A (TSA), attenuates both load- and agonist-induced hypertrophic growth and abolishes the associated activation of autophagy through affecting ATG5 or BECN 1, two essential autophagy effectors (26). HDAC6 is essential for autophagosome-lysosome fusion through binding to polyubiquitinated proteins (27). On the contrary, HDAC7 deactivates autophagy through depressed ERK signaling (28). These pieces of evidence show the importance of HDACs in the pathological progression of PH through autophagy.

The mechanisms underlying the HDAC-related autophagy embrace multiple signaling pathways. In most cases, mTOR inhibition, NF-κB hyperacetylation, ROS accumulation, and the p53 pathway are observed in HDAC-related autophagy signaling (29). Deacetylation of p53 by HDAC enable the accessibility of p53 to its target genes. Additionally, the nuclear export, proteasomal degradation, and co-activator recruitment of p53 are also regulated by HDAC (30). Distinct acetylated residues of p53 are attached by several HATs, increasing the stability and transcriptional activity of p53 binding to sequence-specific target DNA (31). Furthermore, the balance between autophagy and apoptosis is also regulated by p53, providing new insights into the role of p53 in the development of therapeutic drugs of PH.

Multiple inhibitors of HDACs have been developed recently and autophagy activation are frequently observed in the effects of HDAC inhibitor (HDACi) administration. Forkhead box protein O1 (FoxO1) is another important transcript factor in regulating the proliferation and inflammatory signaling of pulmonary artery smooth muscle cells (PASMCs) of PH (32). Inhibiting HDAC with SAHA and TSA activates FoxO1, resulting in mTOR-suppression and ATG-upregulation (33). Acetylation of FoxO1 by SIRT2 prevents its interaction with ATG7, thereby inhibiting autophagy induction (34).

Death-associated protein kinase (DAPK) is a calcium/calmodulin modulated cytoskeleton-associated enzyme, which is closely associated with different MAPKs such as ERK in response to inflammatory apoptotic stimuli (35). Nuclear translocation of DAPK expression is also involved in HDACi-related autophagy activation (36). Additionally, dephosphorylation of DAPK1 at serine 308 by HDAC inhibitor LBH589 promotes autophagy in HCT116 colon cancer cells (37). Studies have shown that DAPK can regulate vascular smooth muscle cell proliferation and migration, promoting vascular inflammation and the development of hypertension diseases (38). Furthermore, vascular calcification is also alleviated by DAPK deficiency (39), which provides a clue regarding the importance of DAPK in PH progression and needs further research.

Sirtuins belong to class III of HDACs. The distinguishing feature of this class of HDACs is that the catalytic activity of the enzyme depends on NAD+ and is regulated by dynamic changes in the NAD+/NADH ratio, suggesting that sirtuins may have evolved into a sensor of energy and redox states in cells. Among the histone-modifying enzymes, the NAD-dependent deacetylase SIRT1 (sirtuin 1) is a particularly well-known modulator of pulmonary hypertension (19). Studies have shown that SIRT1 can regulate the occurrence and process of autophagy and promote the treatment of PH diseases by enhancing the activity of autophagy (40). Specifically, SIRT can promote autophagy by activating key molecules in the autophagy pathway, such as LC3, Beclin-1, and ATG7, or by inhibiting the expression of mTOR, an inhibitor of autophagy. In PH diseases, the expression level of SIRT1 is also regulated. Under hypoxic stress, SIRT1 expression increases, promoting the occurrence of autophagy, which inhibits excessive cell proliferation and hypertrophy of lung vascular wall cells (41). The deacetylase-inactive mutant of SIRT1 disables the induction of autophagy under starvation in mouse embryonic fibroblasts compared with the SIRT1 wild-type gene (34). Histone mark H4K16 is deacetylated by SIRT1 and has great influence on transcriptionally repressing various ATG genes (ULK1/ATG1, ULK3/ATG1, ATG9A/ATG9, LC3/ATG8), leading to the decreased turnover of LC3/ATG8 and autophagic flux (42). Additionally, ATG5, ATG7, and ATG8 bind directly with SIRT1 and can be deacetylated by this binding (43). The stability and function of p53 are also impacted by the enzymatic activity of SIRT1. The degradation of p53 by MDM2 ubiquitination is reversed by SIRT1 expression, which acetylates p53 at lysine 382 and leads to autophagy activation in MCF-7 breast cancer cells (44). Meanwhile, the apoptosis and autophagic cell death of Ishikawa cells are inhibited by SIRT1 through p53 regulation. The subcellular localization of SIRT1 is another influencing factor in autophagy regulation. While SIRT1 limits autophagy in a nucleus under starvation or rapamycin treatment, deacetylation of cytoplasmic proteins by SIRT1 is responsible for autophagy induction (34). These pieces of evidence show that SIRT1 regulates autophagy by both epigenetic and post-translational mechanisms.

Starvation or rapamycin treatment is the well-recognized autophagy-inducing pathway. This canonical process is accompanied by the downregulation of KAT8 and deacetylation of H4K16. Histone acetyltransferase TIP60 activates the acetylation of ULK-1 during starvation-induced autophagy (45). Acetyl coenzyme A (acetyl-CoA), which serves as the donor for acetylation reactions, unavoidably plays an important role in regulating autophagy. High levels of acetyl-CoA inhibit the transcription of ATG7 by hyperacetylation of histone 3 (on K9, K14, and K18) (46). As another well-known autophagy inducer, spermidine is a naturally endogenous polyamine synthesized by diamine putrescine, leading to the inhibition of histone acetyltransferase activity and hypoacetylation of histone 3 (K9, K14, K18) (47). Acetylation of ATG7 promoter is activated and the autophagy-related genes (ATG7, ATG11, ATG15) are increased during spermidine treatment (48).

Generally, acetylation is a common modification and the acetylating regulators have been discovered with abnormal expression in PH. Specifically, acetylation affects autophagy-related genes and proteins such as BECN 1, ATG5, and ATG7, thereby affecting autophagy initiation, autophagosome formation, and the subsequent steps of autophagy. By regulating the expression and function of these key factors, acetylation ultimately affects the role of autophagy in PH. Furthermore, acetylation can also participate in the occurrence and development of pulmonary hypertension through pathways such as regulating endothelial dysfunction, inflammation, mitochondrial energy, and oxidative stress. Therefore, targeting acetylation signaling pathways may represent a promising therapeutic strategy for the treatment of PH.

## 4 Methylation of DNA and histones in the autophagy process of pulmonary hypertension

Methylation refers to the catalytic transfer of methyl groups from active methyl compounds to other compounds. Various methyl compounds can be formed, or certain proteins or nucleic acids can be chemically modified to form methylation products. Within biological systems, methylation is enzymatically catalyzed, involving regulation of gene expression, regulation of protein function, and RNA processing.

### 4.1 DNA methylation

DNA methylation involved in autophagy is regulated by both methyltransferase and demethylase, affecting protein–protein interactions, protein activity, and the interplay with other modifications. There are five members of the DNA methyltransferases (DNMT) family: DNMT1, DNMT2, DNMT3A, DNMT3B, and DNMT3L. DNMT1 catalyzes the methylation of mir-152-3p promoter region and inhibits its expression, which reduces cell viability and inhibits mitophagy progression (49). Additionally, the acetylation of DNMT1 KG-linker is linked to the stability of DNMT1 through USP77 with its UBL1-2 region. The fifth carbon of a cytosine ring in cytosineguanine dinucleotide (CpG) dinucleotides generating 5-methylcytosine (5mC) is transferred with a methyl group by DNMT3A (50), which leads to a stable and heritable DNA methylation mode on target ATGs. Meanwhile, autophagy activation promotes DNMT3A expression, resulting in its combination and transcriptional repression with LC3A, LC3B, and LC3BII genes (51). lncRNA MEG3 promoter methylation is also mediated by DNMT1, which in turn inhibits the ERK/p38/autophagy signaling pathway in bleomycin-induced pulmonary fibrosis (52). DNA methylation of ULK2, ATG5 gene promoter, nitro domain-containing protein 1 (NOR1), death-associated protein kinase (DAPK), and SOX1 also impact the autophagic process (20, 53, 54).

**Figure 4:**
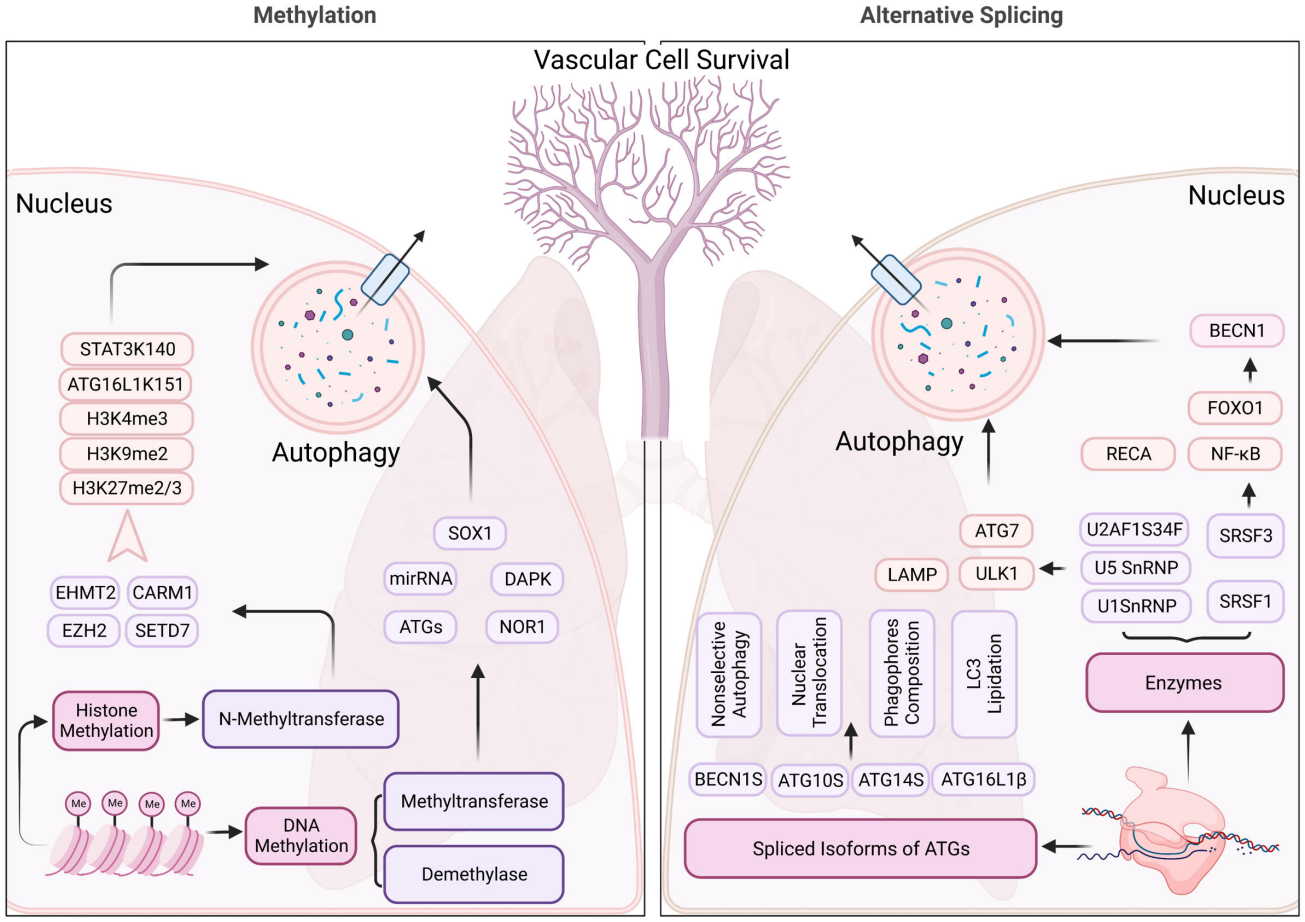
Methylation and alternative splicing located in nucleus regulating the autophagy process of pulmonary hypertension. N-methyltransferase (includes EHMT2, CARM1, EZH2, and SETD7) modulate the autophagy process through methylating histone 3, ATG16L1, and STAT3, contributing to the changes in the structure, function, and expression levels of certain genes, which impact the autophagy process in pulmonary hypertension. Alternative splicing, on the other hand, SnRNPs, and SRSFs regulate the RNA splicing process and can generate different isoforms of proteins involved in autophagy, which may have distinct structures and functions. These mechanisms may contribute to the dysregulation of autophagy in pulmonary hypertension, which is a pathological condition characterized by abnormal cellular remodeling in the lungs.

In PH, DNMT1 and DNMT3B are reported with elevated expression in experimental models induced by Sugen 5416 and hypoxia (55). The DNMT1-HIF-1α- pyruvate dehydrogenase kinase pathway also has an effect on right ventricular fibrosis in MCT-PAH (56). Additionally, bone morphogenetic protein receptor type 2 (BMPR2) promoter is also hypermethylated by switch-independent 3a in human pulmonary arterial smooth muscle cells (57). Furthermore, of therapeutic significance, the present study opens a new avenue for PH treatment by targeting the DNA methylation pathway in pulmonary vessel cells that can modulate the epigenetic landscape of pulmonary vascular genes, and therefore pulmonary vascular and RV remodeling. Given the widespread occurrence of DNA methylation regulation, the direct contact between DNA methylation and autophagy is relatively incomplete and more effort is needed to understand the pathological progression of PH (**Figure 4**).

### 4.2 Histone methylation

Histone methylation mainly occurs on lysine and arginine. Lysine can be monomethylated (me1), dimethylated (me2), or trimethylated (me3), while arginine can be monomethylated (me1), symmetrically dimethylated (me2s), or asymmetrically dimethylated (me2a) on its guanidine group (58). Histone methylation is regulated by protein lysine methyltransferases (PKMTs or PLMTs) and protein arginine methyltransferases (PRMTs), including members of the PRMT family, SET gene family, and non-SET gene family (59, 60). Meanwhile, demethylases mediate the removal of methyl groups from different residues on histones (61). Histone methylation serves as a specific binding site for transcription factors and coregulators. Differential methylation on lysines is able to guide differential activation, causing it to bind to specific promoters. Thus, histone methylation sites form intermediate stations on chromatin that link specific transcription factors or cofactors to downstream gene expression.

Accumulating evidence indicates that histone methylation contributes to the control of cell fate and to the maintenance or suppression of autophagy. Enhancer of zeste homolog 2 (EZH2) gene encodes a histone lysine N-methyltransferase, which methylates histone H3 at position 27 lysine and induces transcriptional repression of the mTOR pathway under serum starvation conditions (62). Metastasis-associated 1 family member 2 (MTA2) recruits EZH2 at specific target gene promoters and catalyzes H3K27me3. In a transverse aortic constriction (TAC)-induced PH mouse model, EZH2 was overexpressed (63). EZH2 promotes vascular smooth muscle cell (VSMC) survival through catalyzing H3K27me2/3, suppressing autophagic cell death by inhibiting the ERK1/2 signaling pathway (64). By inhibiting the expression of EZH2, the proliferation and cell hypertrophy of pulmonary vascular smooth muscle cells can be weakened, and the occurrence of autophagy can be promoted to achieve the effect of treating PH diseases (65, 66). Furthermore, EZH2 methylates STAT3 at Lys180 to activate the STAT3 signaling pathway, which promotes the expression of BCL2 and BCL2L1 and directly inhibits autophagic functions (67). Inhibiting EZH2 with GSK343 or UNC1999 upregulates LC3B and autophagic progression, leading to VSMC loss, enhanced drug sensitivity, and cell death (68, 69). Meanwhile, endogenous knocking-down of EZH2 with RNA interference technology activates cellular senescence-signaling proteins (p16, p53, p14) and inhibits autophagy, which also leads to cell death (70).

H3K9 and H3K4 methylation levels are mainly catalyzed by histone methyltransferases (HMTs) such as euchromatic histone lysine methyltransferase 2 (EHMT2) (71). The dimethylation of H3K9 by EHMT2 represses autophagy gene transcription, which is achieved by the enrichment of EHMT2 on the promoters of ATGs (21). Inhibition of EHMT2 reverses the H3K9me2, resulting in the dissociation of EHMT2 and H3K9me2 from the promoter of ATG6 (72). Autophagy activation also promotes the dissociates EHMT2 from histone H3K9, further decreasing H3K9 dimethylation (73). Additionally, H3K4me3 is also recruited at the promoter region of the damage-regulated autophagy modulator (DRAM) gene upon serum deprivation conditions. DRAM expression is increased by H3K4me3 in serum deprivation-induced autophagy activation. EHMT2 inhibition displays reduced H3K4 methylation and ATGs’ expression in VSMC and various tumors (74). In addition, EHMT2 can also be assembled in the appropriate DNA region, regulating the expression level of autophagy-related nuclear factor transcription factors (75). The methylation levels of H3K4 likely serve as an upstream regulator of autophagy in PH. Specifically, lower methylation levels of EHMT2 are found in hypertensive patients as compared with normotensive subjects (76). Furthermore, emerging evidence suggests a role of DNA methylation in blood physiology, providing a clue regarding the direct link between EHMT2 expression and PH pathology. Overall, in-depth study of the specific mechanism of EHMT2 regulating autophagy will help to further reveal the pathogenesis of PH disease and open up new therapeutic pathways for the treatment of this disease (76).

Coactivator associated arginine methyltransferase 1(CARM1) belongs to PRMTs and increases H3R17me2 levels under glucose deprivation. CARM1 regulates autophagy through transcriptionally coactivating ATGs and lysosomal genes with transcription factor EB (TFEB) (77). Stem cell factor (SCF) E3 ubiquitin ligase is responsible for degrading and destabilizing CARM1 when autophagy is depressed by AMPK inhibition. CARM1-mediated histone arginine methylation seems to be a critical nuclear event in the regulation of autophagy and targeting the AMPK-CARM1 signaling pathway may abrogate the pathological progression in autophagy-related diseases. In addition, CARM1 can also regulate the expression levels of ATGs and nuclear factors by assembling in the appropriate DNA region, playing a regulatory role in multiple cellular responses such as nuclear factors, transcription factors, and cell signaling pathways (78). The homocysteine metabolism pathway is also reported to be regulated by CARM1, which is a risk factor for vascular disease (79). This evidence provides a clue regarding the importance of CARM1 in autophagy regulation of PH disease.

SET domain containing 7 (SETD7) belongs to the SET gene family, which is histone lysine methyltransferase containing highly conserved SET domains. SETD7 methylates ATG16L1 at lysine 151, impairing the ATG16L1-ATG12-ATG5 complex and autophagy progression. This methylation of ATG16L1 is reversed by lysine demethylase 1A (LSD1/KDM1A), inhibiting hypoxia/reoxygenation-induced cardiomyocyte apoptosis (80). Furthermore, methylated ATG16L1 at lysine 151 prevents its phosphorylation at S139, which is critical for autophagy maintenance (81). Moreover, SETD7 also dimethylates STAT3 at Lys 140, which prevents its phosphorylation at Tyr705 and inhibits STAT3 activity (82). STAT3 is critical for transcriptionally upregulating HIF1a, which is stabilized and induces autophagy under hypoxic conditions in PH (83). Located at 4q28.3-31.23, SETD7 deletion may impact lung vascular malformation leading to PH (84). The direct link between SETD7 regulation in the autophagy process of PH still needs more investigation and may play a significant role in the development of PH therapeutic drugs. Above all, histone methylation has been discovered with widespread existence in numerous human diseases. Many methylation regulators exhibit abnormal phenotype expression and have effects on PH progression. Studies of G9a and EZH2 in PH indicate that histone methylation plays an essential role in PASMC proliferation of PH. Specifically, increased levels of H3K9me2 and H3K9me3 contribute to impaired autophagic flux and the exacerbation of PH pathogenesis, while targeting these histone modifications has shown promise as a therapeutic strategy for PH.

## 5 RNA alternative splicing is another important regulatory machinery affecting autophagy

### 5.1 RNA alternative splicing regulatory enzymes

Transcription and translation make up the entire process of gene expression, and RNA splicing is a crucial step in this process. Due to the complexity and diversity of RNA splicing, alternative splicing is an important contributor to proteome richness. Small nuclear ribonucleoproteins (SnRNPs) and splicing regulatory factors (SRSF proteins, hnRNPs, RBPs) play important roles in finishing the transcription, and are involved in numerous human diseases including PH through ATGs regulation (**Figure 4**).

U1 snRNP overexpression induced by presenilin 1 affects autophagy by impairment of lysosomal biogenesis and autophagosome-lysosome fusion, which in turn affects the turnover of these peptides and accelerates neuronal cell death (85). Knocking-down the pre-mRNA splicing factor (PRPF8), which is a component of U5 snRNP and central module of the spliceosome, leads to enhanced skipped splicing of exon 22 and exon 23 of ULK1 at the mRNA level. This splicing process abrogates mitophagosome formation and the clearance of mitochondria (86, 87). U2AF1S34F mutant protein of the pre-mRNA splicing factor U2AF alters the 3′end formation of mRNAs through distal cleavage and polyadenylation sites, which results in ATG7’ depression (88).

SRSF1 and SRSF3 belong to the splicing regulatory factor family and inhibit autophagosome formation through their effect on the transcription factor (RELA proto-oncogene, NF-κB subunit, forkhead box O1)-BECN1 pathway in lung adenocarcinoma cells (89). The splicing of the long isoform of Bcl-x that interacts with Beclin1 is also promoted by SRSF1, thereby dissociating the Beclin1-PIK3C3 complex (89). Similarly, the 3′UTR of BECN1 is also regulated by binding with hnRNPA1, the effects of which remain to be established in PH (90).

Alternative splicing of the autophagy gene ATG5 has been shown to regulate apoptosis of pulmonary artery smooth muscle cells (PASMCs) in PH. The inclusion of a specific exon in the ATG5 mRNA, which is promoted by the splicing factor SRSF1, results in increased autophagy activity and decreased PASMC apoptosis (91).

SRSF3 has been shown to promote PH by regulating the expression of the autophagy gene ATG16L1. Specifically, SRSF3 promotes the inclusion of a specific exon in the ATG16L1 mRNA, resulting in increased autophagy activity and enhanced vascular remodeling (92). Conversely, other splicing factors such as RBM4 (RNA binding motif protein 4) can inhibit PH by promoting the exclusion of the same exon from the ATG16L1 mRNA, leading to decreased autophagy activity and attenuated vascular remodeling (93).

Muscleblind like splicing regulators (MBNLs) modulate alternative splicing through exon skipping. MBNL1 and MBNL2 interact directly with the rubicon autophagy regulator (RUBCN) through RNA binding, leading to the exon skipping of RUBCN and increases of autophagy (94, 95). Additionally, utilizing the antiautophagic drug chloroquine is sufficient to upregulate MBNL1 and 2 proteins (96). Chloroquine has been proven to be efficient in experimental mouse PH models, but the expression and function of MBNLs deserve a deeper study.

Alternative splicing regulation is an emerging mechanism of gene expression control, which affects protein translation and function by altering the splicing patterns of RNA. Alternative splicing of BMPR2 by SRSF2 has a role in PH penetration and makes developing PH more likely (97). Specifically, this regulatory mechanism can affect the transcription and alternative splicing of genes related to autophagy, thereby influencing the progression and efficiency of autophagy. These studies suggest that alternative splicing regulation plays a crucial role in autophagy in pulmonary hypertension. Therefore, a deeper understanding of the mechanisms underlying the alternative splicing regulation of autophagy in pulmonary hypertension is crucial for drug development and treatment.

### 5.2 Alternative spliced isoforms of ATGs affect the autophagic process differentially

Alternative splicing creates multiple isoforms of ATGs which may differ from the primary subunit in structure and function. An additional splice variant of BECN1 called BECN1 short isoform (BECN1S) that lacks both exon 10 and 11 has been reported in multiple cell types. This different BECN1 variant exhibits distinct functions in controlling autophagy compared to BECN1. Overexpression of BECN1S abrogates starvation-induced autophagy in AML cells as a negative regulator (98). Whereas BECN1S is useless in macroautophagy, it could on the other hand support mitophagy (99). These phenomena point out its effect in non-selective autophagy and selective mitophagy. Studies have shown that the ratio of BECN-1S to BECN-1 is altered in PH, with a shift towards the production of BECN-1. This shift has been shown to impair autophagy flux and lead to endothelial cell dysfunction. In addition to BECN-1, other autophagy-related genes have also been shown to undergo alternative splicing in PH (100). For example, the splicing of ATG5, another key autophagy regulator, has been shown to be altered in PH. This altered splicing leads to the production of a truncated isoform of ATG5, which has been shown to impair autophagy flux and lead to endothelial cell dysfunction (101).

Likewise, ATG10S, which is distinct from ATG10 at 36 amino acids at the N terminus promotes autophagy completion that degrades HCV subgenomic and genomic replicons. Meanwhile, the absence at Cys44 of ATG10S makes it possible for its nuclear translocation and combination with interferon lambda 2 (IL28A), which mediate the clearance of viral infections through autophagy (102).

ATG14 is another essential core component in the early steps of autophagy (103). ATG14S is a short variant of ATG14 which lacks the cysteine repeats-containing domain required for autophagosome-endolysosome fusion. Moreover, this oligomerization domain is not functional in the PtdIns3K nucleation complex formation, which retains the function of ATG14L and ATG14S in phagophores composition (104).

ATG7 is required for LC3 lipidation for its E1-like enzyme activity. ATG7(2) is distinct from ATG7(1) at a deprived exon of 27 amino acids. Additionally, ATG7(2) lacks the region required for homodimerization and the binding between ATG7 and LC3, thus inhibiting the lipidation of LC3 (105).

ATG16L1 participates in autophagy regulation in multiple aspects, like binding with ATG5 through its N-terminal region and engaging in LC3 lipidation (106). Three slicing variants of ATG16L1 have been discovered so far and ATG16L1β (absence of exon 9) contains a unique β-isoform lipid-binding region, which is indispensable for LC3 lipidation under endosomal stress (107).

Lysosomal-associated membrane protein 2B (LAMP2B) is abundant in lysosomes and is indispensable in autophagosome fusion with late endosomes/lysosomes. The differences between LAMP2C and LAMP2B are located at the lysosomal carboxyl-terminal transmembrane region and the cytoplasmic short tail. Meanwhile, LAMP2C affects DNautophagy and RNautophagy through interacting with RBPs (108). LAMP2C is reported to interact with RBPs and nucleic acid proteins such as histone 1, suggesting a role in the uptake and degradation of RNA and DNA molecules within the lysosome, processes known as DNautophagy and RNautophagy (108).

To summarize, studies have shown that alternative splicing variants of ATGs may be associated with the occurrence and development of PH. Specifically, some alternative splicing variants of autophagy genes increase the expression levels in pulmonary vascular smooth muscle cells, leading to enhanced intracellular autophagy processes and promoting the development of PH. In addition, certain alternative splicing variants may also affect PH through pathways such as cell apoptosis, proliferation, and metabolism. However, the research on the relationship between alternative splicing variants of ATGs and PH is still in its early stages and requires further investigation.

### 5.3 MirRNAs

MirRNAs are small (21–25 nucleotides) single stranded RNAs that bind to complementary nascent mRNAs and render them susceptible to degradation prior to translation, thereby inhibiting the expression of specific target genes. RNA interference is an epigenetic mechanism of gene regulation that occurs post-transcriptionally. MirRNAs plays an important role in autophagy regulation. For example, some microRNAs can inhibit the expression of autophagy-related genes, thus inhibiting the autophagy process, while some long non-coding RNAs can participate in the induction and execution process of autophagy. For example, MirRNA-10a, MirRNA-20a, and MirRNA-885-3p target RB1CC1, ULK1, and ULK2, respectively, and impact the induction of autophagy (109–111). MirRNA-34a, MirRNA-195, MirRNA-30a, and MirRNA-30a bind ATG9, ATG14, BECN1, and UVRAG, respectively, in the nucleation process of autophagy (112–114). The elongation of autophagy phospholipid membrane is regulated by ATG4, ATG5, ATG7, ATG10, ATG12, ATG16, LC3, and SQSTM1, which are also affected by MirRNA-101, MirRNA-30a, MirRNA-17, MirRNA-519a, MirRNA-30d, MirRNA-519a, MirRNA-204, and MirRNA-17 (111, 115–119). This evidence elucidates that MirRNA participates in regulating the whole process of autophagy and has an effect on the completion of autophagy.

In recent years, small RNAs have gained more and more attention and numerous studies have made progress in the regulation of small RNAs in PH. CircSIRT1 impacts the biological behavior of hypoxia-stimulated PASMC *via* modulating the Mir-145-5p/Akt3 pathway (120). The circ-calm4/Purb/BECN1 signal axis is involved in the occurrence of hypoxia-induced PASMCs autophagy, and the novel regulatory mechanisms and signals transduction pathways in PASMC autophagy are induced by hypoxia (121). LncRNA-GAS5/Mir-382-3p axis promotes autophagy and inhibits pulmonary artery remodeling. MirRNA-874-5P regulates autophagy and proliferation in PASMCs by targeting Sirtuin3 (41). Mir204 enhances autophagy and promotes endothelial-mesenchymal in hypoxia PH (122). Targeting this MirRNA has been shown to restore autophagy and improve disease symptoms in animal models of PH. These non-coding RNAs modulate the ATGs with relatively simple and direct mechanisms through binding and inhibiting the target genes. These molecules have emerged as attractive targets for PH therapy due to their ability to modulate various signaling pathways involved in disease progression, including autophagy.

Targeting MirRNAs that regulate autophagy is a potential personalized therapy. Currently, research on the mechanism of MirRNA regulating autophagy mainly focuses on the following aspects: identifying and describing new regulatory factors, exploring the molecular mechanisms of regulatory factors, and studying the role of regulatory factors in PH. These studies are of great significance for us to deepen our understanding of autophagy regulation mechanisms, develop treatment methods for autophagy-related diseases, and so on. However, it should be noted that there are still many unknowns about the mechanism of MirRNA regulating autophagy which require further research and exploration.

## 6 Conclusions and future perspectives

PH is a type of chronic life-threatening cardiovascular disease that is difficult to cure clinically, the pathogenesis of which is complex and not yet fully understood. Epigenetic regulation affects the expression, stability, and function of ATGs and plays important roles in the development of PH. Certain epigenetic modification patterns (such as DNA methylation and histone modification) are reported with abnormal dysfunction in patients with pulmonary hypertension, leading to dysregulated gene expression and promoting pathological processes such as pulmonary vessel tension and cell proliferation. In this review, mounting evidence has demonstrated that crosstalk between epigenetic modifications and autophagy is related to the initiation and progression of PH. Acetylation regulated by HDACs and HATs occurs in multiple signaling pathways which directly or indirectly act on ATGs, which can either promote or inhibit autophagic activity depending on the specific lysine residues targeted and the acetyltransferase/deacetylase enzymes involved. In preclinical studies, HDAC inhibition has been shown to attenuate PH by enhancing autophagy in both endothelial cells and smooth muscle cells. This effect is mediated by the acetylation of histones upstream of autophagy-related genes such as BECN-1 and LC3 (123). A HDAC inhibitor is one potential approach for personalized treatment strategies targeting epigenetic regulation of autophagy. However, more research is needed to determine whether HDACi are a viable therapeutic option for human PH (124). Understanding the precise mechanisms by which acetylation regulates autophagy is an active area of research, with potential therapeutic implications for diseases associated with autophagy in PH. Targeting acetylation and deacetylation pathways may offer new strategies for enhancing or inhibiting autophagy in disease states, although further preclinical and clinical studies are needed to evaluate their efficacy and safety.

Additionally, the aberrant methylation of histones and DNA in nucleus serves as another important pattern contributing to autophagy dysfunction in various diseases, such as cancer, neurodegenerative disorders, metabolic disorders, and PH. Hypermethylation of ATGs leads to decreased autophagic activity and increased growth. However, the relationship between methylation and autophagy is complex and context-dependent, and more research is needed to fully understand the underlying mechanisms. Furthermore, it remains unclear whether targeting DNA methylation could be a viable therapeutic approach for modulating autophagy in PH. Overall, the study of methylation regulation of autophagy presents an exciting avenue for future research, with potential implications for the development of novel therapeutic strategies.

Alternative splicing has effects on autophagy in PH through producing multiple protein isoforms with distinct structures. It can generate both pro-autophagic and anti-autophagic isoforms with opposing functions. Considering the complexity of alternative splicing, more research is needed to elucidate the exact molecular pathways involved. Overall, the study of alternative splicing regulation of autophagy presents a promising area for future research. With a better understanding of the complex interplay between alternative splicing and autophagy, researchers may be able to uncover new targets for drug development and improve our ability to treat PH involving autophagy dysfunction.

As for now, the clinical treatment of autophagy in PH has progressed rapidly, which will be elaborated in depth below. First, cytokine inhibitors (125): currently, research has found that one of the therapeutic strategies for autophagy is to regulate autophagy by regulating cytokine levels. Specifically, the evaluation and regulation of cytokines such as TGF-β, BMP, and PDGF in PH diseases has become a research hotspot. Treatments that target these cytokines, such as the use of specific inhibitors, play an important role in restorative enhancement therapy for autophagy (126). Second, the development of agonists and inhibitors (127): studies have found that treatment targeting specific autophagy modulators can promote cardiovascular recovery and reduce symptoms of PH. There are currently many autophagy regulators being developed, including autophagy agonists and inhibitors. Studies of autophagy agonists in the treatment of PH have shown that some autophagy agonists can reduce symptoms in patients with PH by enhancing the activity of autophagy (128). For example, according to one study, Sirt1 agonists reduce symptoms in rats with PH, reducing the pulmonary vascular resistance and right ventricular weight index by promoting autophagy. Similarly, another study showed that a drug called RapaLink-1 that utilizes autophagy pathway drug delivery can reduce pathological damage and thymus peptide secretion in mice. These findings suggest that autophagy agonists may become a novel drug for the treatment of PH. Acting as an autophagy inhibitor, chloroquine has a significant therapeutic effect in patients with PH. A randomized, double-blind, placebo-controlled trial of chloroquine in the treatment of PH showed that chloroquine significantly reduced clinical symptoms and blood biochemical parameters and increased exercise tolerance and lung function (129). These data suggest that chloroquine, as a drug regulating autophagy, may be a good prospect in the treatment of PH. In general, chloroquine will become a major trend in the development of autophagy agonists in the treatment of PH. Its complex mechanism of action and definite efficacy provides a new treatment option for patients with PH. However, chloroquine needs further clinical studies to support it as the first-line drug for the treatment of PH (9). As such, personalized treatment strategies targeting these pathways may be effective in improving outcomes for patients with PH.

Above all, epigenetic regulation of autophagy plays an important role in the pathogenesis of PH. Researchers are currently exploring how to use epigenetic regulation and autophagy to treat PH. Inhibiting autophagy through drug intervention can successfully alleviate the severity of PH. In addition, some epigenetic modification repair technologies are also under development to restore damaged gene expression patterns. Further clinical and translational research is needed to optimize screening, diagnostics (including genetic testing), establish therapies, and tailor therapy to treat the underlying pathological processes in PH patients. With the advancement of technology and knowledge, we can expect more effective and personalized treatment options to emerge focusing on epigenetic regulation and autophagy.

## Author contributions

MM and SS designed and wrote the manuscript. XL and JYL sourced the relevant literature. JiL and WZ helped write the manuscript with constructive discussions. HL, JXL, and BZ developed the concepts, design, definition of intellectual content, and undertook the literature search. All authors read and approved the final version of the manuscript.

## References

[1] Li X He S Ma B. Autophagy and autophagy-related proteins in cancer. Mol Cancer. (2020) 19(1):12. doi: 10.1186/s12943-020-1138-4 31969156 PMC6975070

[2] Dikic I Elazar Z. Mechanism and medical implications of mammalian autophagy. Nat Rev Mol Cell Biol (2018) 19(6):349–64. doi: 10.1038/s41580-018-0003-4 29618831

[3] Fraidenburg DR Yuan JX. Hungry for more: autophagy in the pathogenesis of pulmonary arterial hypertension. Circ Res (2013) 112(8):1091–3. doi: 10.1161/CIRCRESAHA.113.301247 PMC3728159 23580770

[4] Klionsky DJ Cregg JM Dunn WA Jr. Emr SD Sakai Y Sandoval IV. A unified nomenclature for yeast autophagy-related genes. Dev Cell (2003) 5(4):539–45. doi: 10.1016/s1534-5807(03)00296-x 14536056

[5] Daniell H Mangu V Yakubov B Park J Habibi P Shi Y. Investigational new drug enabling angiotensin oral-delivery studies to attenuate pulmonary hypertension. Biomaterials (2020) 233:119750. doi: 10.1016/j.biomaterials.2019.119750 31931441 PMC7045910

[6] Simonneau G Montani D Celermajer DS Denton CP Gatzoulis MA Krowka M. Haemodynamic definitions and updated clinical classification of pulmonary hypertension. Eur Respir J (2019) 53(1):1801913. doi: 10.1183/13993003.01913-2018 30545968 PMC6351336

[7] Hoeper MM Humbert M Souza R Idrees M Kawut SM Sliwa-Hahnle K. A global view of pulmonary hypertension. Lancet Respir Med (2016) 4(4):306–22. doi: 10.1016/S2213-2600(15)00543-3 26975810

[8] Humbert M Guignabert C Bonnet S Dorfmuller P Klinger JR Nicolls MR. Pathology and pathobiology of pulmonary hypertension: state of the art and research perspectives. Eur Respir J (2019) 53(1):1801887. doi: 10.1183/13993003.01887-2018 30545970 PMC6351340

[9] Long L Yang X Southwood M Lu J Marciniak SJ Dunmore BJ. Chloroquine prevents progression of experimental pulmonary hypertension *via* inhibition of autophagy and lysosomal bone morphogenetic protein type II receptor degradation. Circ Res (2013) 112(8):1159–70. doi: 10.1161/CIRCRESAHA.111.300483 23446737

[10] Nakahira K Cloonan SM Mizumura K Choi AM Ryter SW. Autophagy: a crucial moderator of redox balance, inflammation, and apoptosis in lung disease. Antioxid Redox Signal (2014) 20(3):474–94. doi: 10.1089/ars.2013.5373 PMC3894710 23879400

[11] Ornatowski W Lu Q Yegambaram M Garcia AE Zemskov EA Maltepe E. Complex interplay between autophagy and oxidative stress in the development of pulmonary disease. Redox Biol (2020) 36:101679. doi: 10.1016/j.redox.2020.101679 32818797 PMC7451718

[12] Zhang CF Zhao FY Xu SL Liu J Xing XQ Yang J. Autophagy in pulmonary hypertension: emerging roles and therapeutic implications. J Cell Physiol (2019) 234(10):16755–67. doi: 10.1002/jcp.28531 30932199

[13] Chichger H Rounds S Harrington EO. Endosomes and autophagy: regulators of pulmonary endothelial cell homeostasis in health and disease. Antioxid Redox Signal (2019) 31(13):994–1008. doi: 10.1089/ars.2019.7817 31190562 PMC6765061

[14] Mao J Ma L. Research progress on the mechanism of phenotypic transformation of pulmonary artery smooth muscle cells induced by hypoxia. Zhejiang Da Xue Xue Bao Yi Xue Ban. (2023) 51(6):750–7. doi: 10.3724/zdxbyxb-2022-0282 PMC10262008 36915980

[15] Chen R Jiang M Li B Zhong W Wang Z Yuan W. The role of autophagy in pulmonary hypertension: a double-edge sword. Apoptosis (2018) 23(9-10):459–69. doi: 10.1007/s10495-018-1477-4 30117075

[16] Bu S Singh KK. Epigenetic regulation of autophagy in cardiovascular pathobiology. Int J Mol Sci (2021) 22(12):6544. doi: 10.3390/ijms22126544 34207151 PMC8235464

[17] Wang K Chen Y Zhang P Lin P Xie N Wu M. Protective features of autophagy in pulmonary infection and inflammatory diseases. Cells (2019) 8(2):123. doi: 10.3390/cells8020123 30717487 PMC6406971

[18] Baek SH Kim KI. Epigenetic control of autophagy: nuclear events gain more attention. Mol Cell (2017) 65(5):781–5. doi: 10.1016/j.molcel.2016.12.027 28257699

[19] Chelladurai P Boucherat O Stenmark K Kracht M Seeger W Bauer UM. Targeting histone acetylation in pulmonary hypertension and right ventricular hypertrophy. Br J Pharmacol (2021) 178(1):54–71. doi: 10.1111/bph.14932 31749139

[20] Hu LF. Epigenetic regulation of autophagy. Adv Exp Med Biol (2019) 1206:221–36. doi: 10.1007/978-981-15-0602-4_11 31776988

[21] Lapierre LR Kumsta C Sandri M Ballabio A Hansen M. Transcriptional and epigenetic regulation of autophagy in aging. Autophagy (2015) 11(6):867–80. doi: 10.1080/15548627.2015.1034410 PMC4502732 25836756

[22] Feng W Wang J Yan X Zhang Q Chai L Wang Q. ERK/Drp1-dependent mitochondrial fission contributes to HMGB1-induced autophagy in pulmonary arterial hypertension. Cell Prolif. (2021) 54(6):e13048. doi: 10.1111/cpr.13048 33948998 PMC8168414

[23] Pratt R Dufrenoy J. Practical three-hour and two-hour cylinder-plate assays for penicillin. Nature (1947) 159(4043):576. doi: 10.1038/159576a0 20297075

[24] Zhao L Chen CN Hajji N Oliver E Cotroneo E Wharton J. Histone deacetylation inhibition in pulmonary hypertension: therapeutic potential of valproic acid and suberoylanilide hydroxamic acid. Circulation (2012) 126(4):455–67. doi: 10.1161/CIRCULATIONAHA.112.103176 PMC3799888 22711276

[25] Cavasin MA Stenmark KR McKinsey TA. Emerging roles for histone deacetylases in pulmonary hypertension and right ventricular remodeling (2013 grover conference series). Pulm Circ (2015) 5(1):63–72. doi: 10.1086/679700 25992271 PMC4405717

[26] Cao DJ Wang ZV Battiprolu PK Jiang N Morales CR Kong Y. Histone deacetylase (HDAC) inhibitors attenuate cardiac hypertrophy by suppressing autophagy. Proc Natl Acad Sci U S A. (2011) 108(10):4123–8. doi: 10.1073/pnas.1015081108 PMC3053983 21367693

[27] Zhang J Zhong Q. Histone deacetylase inhibitors and cell death. Cell Mol Life Sci (2014) 71(20):3885–901. doi: 10.1007/s00018-014-1656-6 PMC4414051 24898083

[28] Ahn MY Yoon JH. Histone deacetylase 7 silencing induces apoptosis and autophagy in salivary mucoepidermoid carcinoma cells. J Oral Pathol Med (2017) 46(4):276–83. doi: 10.1111/jop.12560 28178760

[29] Mrakovcic M Bohner L Hanisch M Frohlich LF. Epigenetic targeting of autophagy *via* HDAC inhibition in tumor cells: role of p53. Int J Mol Sci (2018) 19(12):3952. doi: 10.3390/ijms19123952 30544838 PMC6321134

[30] Sykes SM Stanek TJ Frank A Murphy ME McMahon SB. Acetylation of the DNA binding domain regulates transcription-independent apoptosis by p53. J Biol Chem (2009) 284(30):20197–205. doi: 10.1074/jbc.M109.026096 PMC2740446 19494119

[31] Gu W Roeder RG. Activation of p53 sequence-specific DNA binding by acetylation of the p53 c-terminal domain. Cell (1997) 90(4):595–606. doi: 10.1016/s0092-8674(00)80521-8 9288740

[32] Savai R Al-Tamari HM Sedding D Kojonazarov B Muecke C Teske R. Pro-proliferative and inflammatory signaling converge on FoxO1 transcription factor in pulmonary hypertension. Nat Med (2014) 20(11):1289–300. doi: 10.1038/nm.3695 25344740

[33] Ellis L Bots M Lindemann RK Bolden JE Newbold A Cluse LA. The histone deacetylase inhibitors LAQ824 and LBH589 do not require death receptor signaling or a functional apoptosome to mediate tumor cell death or therapeutic efficacy. Blood (2009) 114(2):380–93. doi: 10.1182/blood-2008-10-182758 PMC4580966 19383971

[34] Lee IH Cao L Mostoslavsky R Lombard DB Liu J Bruns NE. A role for the NAD-dependent deacetylase Sirt1 in the regulation of autophagy. Proc Natl Acad Sci U S A. (2008) 105(9):3374–9. doi: 10.1073/pnas.0712145105 PMC2265142 18296641

[35] Gandesiri M Chakilam S Ivanovska J Benderska N Ocker M Di Fazio P. DAPK plays an important role in panobinostat-induced autophagy and commits cells to apoptosis under autophagy deficient conditions. Apoptosis (2012) 17(12):1300–15. doi: 10.1007/s10495-012-0757-7 23011180

[36] Zhang J Ng S Wang J Zhou J Tan SH Yang N. Histone deacetylase inhibitors induce autophagy through FOXO1-dependent pathways. Autophagy (2015) 11(4):629–42. doi: 10.1080/15548627.2015.1023981 PMC4502718 25919885

[37] Fulda S Kufer MU Meyer E van Valen F Dockhorn-Dworniczak B Debatin KM. Sensitization for death receptor- or drug-induced apoptosis by re-expression of caspase-8 through demethylation or gene transfer. Oncogene (2001) 20(41):5865–77. doi: 10.1038/sj.onc.1204750 11593392

[38] Usui T Sakatsume T Nijima R Otani K Kazama K Morita T. Death-associated protein kinase 3 mediates vascular structural remodelling *via* stimulating smooth muscle cell proliferation and migration. Clin Sci (Lond). (2014) 127(8):539–48. doi: 10.1042/CS20130591 24814693

[39] Li KX Du Q Wang HP Sun HJ. Death-associated protein kinase 3 deficiency alleviates vascular calcification *via* AMPK-mediated inhibition of endoplasmic reticulum stress. Eur J Pharmacol (2019) 852:90–8. doi: 10.1016/j.ejphar.2019.03.007 30851272

[40] Lai YC Tabima DM Dube JJ Hughan KS Vanderpool RR Goncharov DA. SIRT3-AMP-Activated protein kinase activation by nitrite and metformin improves hyperglycemia and normalizes pulmonary hypertension associated with heart failure with preserved ejection fraction. Circulation (2016) 133(8):717–31. doi: 10.1161/CIRCULATIONAHA.115.018935 PMC4766041 26813102

[41] Zhang L Ma C Wang X Bai J He S Zhang J. MicroRNA-874-5p regulates autophagy and proliferation in pulmonary artery smooth muscle cells by targeting sirtuin 3. Eur J Pharmacol (2020) 888:173485. doi: 10.1016/j.ejphar.2020.173485 32805255

[42] Fullgrabe J Klionsky DJ Joseph B. The return of the nucleus: transcriptional and epigenetic control of autophagy. Nat Rev Mol Cell Biol (2014) 15(1):65–74. doi: 10.1038/nrm3716 24326622

[43] Morselli E Marino G Bennetzen MV Eisenberg T Megalou E Schroeder S. Spermidine and resveratrol induce autophagy by distinct pathways converging on the acetylproteome. J Cell Biol (2011) 192(4):615–29. doi: 10.1083/jcb.201008167 PMC3044119 21339330

[44] Wang J Kim TH Ahn MY Lee J Jung JH Choi WS. Sirtinol, a class III HDAC inhibitor, induces apoptotic and autophagic cell death in MCF-7 human breast cancer cells. Int J Oncol (2012) 41(3):1101–9. doi: 10.3892/ijo.2012.1534 22751989

[45] Lin SY Li TY Liu Q Zhang C Li X Chen Y. Protein phosphorylation-acetylation cascade connects growth factor deprivation to autophagy. Autophagy (2012) 8(9):1385–6. doi: 10.4161/auto.20959 PMC3442885 22717509

[46] Eisenberg T Schroeder S Andryushkova A Pendl T Kuttner V Bhukel A. Nucleocytosolic depletion of the energy metabolite acetyl-coenzyme a stimulates autophagy and prolongs lifespan. Cell Metab (2014) 19(3):431–44. doi: 10.1016/j.cmet.2014.02.010 PMC3988959 24606900

[47] Ghosh I Sankhe R Mudgal J Arora D Nampoothiri M. Spermidine, an autophagy inducer, as a therapeutic strategy in neurological disorders. Neuropeptides (2020) 83:102083. doi: 10.1016/j.npep.2020.102083 32873420

[48] Eisenberg T Knauer H Schauer A Buttner S Ruckenstuhl C Carmona-Gutierrez D. Induction of autophagy by spermidine promotes longevity. Nat Cell Biol (2009) 11(11):1305–14. doi: 10.1038/ncb1975 19801973

[49] Deng Z Yao J Xiao N Han Y Wu X Ci C. DNA Methyltransferase 1 (DNMT1) suppresses mitophagy and aggravates heart failure *via* the microRNA-152-3p/ETS1/RhoH axis. Lab Invest. (2022) 102(8):782–93. doi: 10.1038/s41374-022-00740-8 35149775

[50] Ehrlich M Wang RY. 5-methylcytosine in eukaryotic DNA. Science (1981) 212(4501):1350–7. doi: 10.1126/science.6262918 6262918

[51] Gonzalez-Rodriguez P Cheray M Fullgrabe J Salli M Engskog-Vlachos P Keane L. The DNA methyltransferase DNMT3A contributes to autophagy long-term memory. Autophagy (2021) 17(5):1259–77. doi: 10.1080/15548627.2020.1816664 PMC8143216 32876528

[52] Ting L Feng Y Zhou Y Tong Z Dong Z. IL-27 induces autophagy through regulation of the DNMT1/lncRNA MEG3/ERK/p38 axis to reduce pulmonary fibrosis. Respir Res (2023) 24(1):67. doi: 10.1186/s12931-023-02373-x 36869378 PMC9985266

[53] Motoo I Nanjo S Ando T Yamashita S Ushijima T Yasuda I. Methylation silencing of ULK2 *via* epithelial-mesenchymal transition causes transformation to poorly differentiated gastric cancers. Gastric Cancer. (2022) 25(2):325–35. doi: 10.1007/s10120-021-01250-0 34554345

[54] Chen YC Lin IC Su MC Hsu PY Hsiao CC Hsu TY. Autophagy impairment in patients with obstructive sleep apnea modulates intermittent hypoxia-induced oxidative stress and cell apoptosis *via* hypermethylation of the ATG5 gene promoter region. Eur J Med Res (2023) 28(1):82. doi: 10.1186/s40001-023-01051-4 36805797 PMC9936724

[55] Jacob C Kitagawa A Signoretti C Dzieciatkowska M D’Alessandro A Gupte A. Mediterranean G6PD variant mitigates expression of DNA methyltransferases and right heart pressure in experimental model of pulmonary hypertension. J Biol Chem (2022) 298(12):102691. doi: 10.1016/j.jbc.2022.102691 36372233 PMC9731845

[56] Tian L Wu D Dasgupta A Chen KH Mewburn J Potus F. Epigenetic metabolic reprogramming of right ventricular fibroblasts in pulmonary arterial hypertension: a pyruvate dehydrogenase kinase-dependent shift in mitochondrial metabolism promotes right ventricular fibrosis. Circ Res (2020) 126(12):1723–45. doi: 10.1161/CIRCRESAHA.120.316443 PMC7274861 32216531

[57] Bisserier M Mathiyalagan P Zhang S Elmastour F Dorfmuller P Humbert M. Regulation of the methylation and expression levels of the BMPR2 gene by SIN3a as a novel therapeutic mechanism in pulmonary arterial hypertension. Circulation (2021) 144(1):52–73. doi: 10.1161/CIRCULATIONAHA.120.047978 34078089 PMC8293289

[58] Greer EL Shi Y. Histone methylation: a dynamic mark in health, disease and inheritance. Nat Rev Genet (2012) 13(5):343–57. doi: 10.1038/nrg3173 PMC4073795 22473383

[59] Murray K. The occurrence of epsilon-N-Methyl lysine in histones. Biochemistry (1964) 3:10–5. doi: 10.1021/bi00889a003 14114491

[60] Byvoet P Shepherd GR Hardin JM Noland BJ. The distribution and turnover of labeled methyl groups in histone fractions of cultured mammalian cells. Arch Biochem Biophys (1972) 148(2):558–67. doi: 10.1016/0003-9861(72)90174-9 5063076

[61] Mosammaparast N Shi Y. Reversal of histone methylation: biochemical and molecular mechanisms of histone demethylases. Annu Rev Biochem (2010) 79:155–79. doi: 10.1146/annurev.biochem.78.070907.103946 20373914

[62] Wei FZ Cao Z Wang X Wang H Cai MY Li T. Epigenetic regulation of autophagy by the methyltransferase EZH2 through an MTOR-dependent pathway. Autophagy (2015) 11(12):2309–22. doi: 10.1080/15548627.2015.1117734 PMC4835210 26735435

[63] Aljubran SA Cox R Jr. Tamarapu Parthasarathy P Kollongod Ramanathan G Rajanbabu V Bao H. Enhancer of zeste homolog 2 induces pulmonary artery smooth muscle cell proliferation. PloS One (2012) 7(5):e37712. doi: 10.1371/journal.pone.0037712 22662197 PMC3360676

[64] Li R Yi X Wei X Huo B Guo X Cheng C. EZH2 inhibits autophagic cell death of aortic vascular smooth muscle cells to affect aortic dissection. Cell Death Dis (2018) 9(2):180. doi: 10.1038/s41419-017-0213-2 29416002 PMC5833461

[65] Wang Y Huang XX Leng D Li JF Liang Y Jiang T. Effect of EZH2 on pulmonary artery smooth muscle cell migration in pulmonary hypertension. Mol Med Rep (2021) 23(2):129. doi: 10.3892/mmr.2020.11768 33313943 PMC7751464

[66] Shi ZL Fang K Li ZH Ren DH Zhang JY Sun J. EZH2 inhibition ameliorates transverse aortic constriction-induced pulmonary arterial hypertension in mice. Can Respir J (2018) 2018:9174926. doi: 10.1155/2018/9174926 29854032 PMC5960552

[67] Kim E Kim M Woo DH Shin Y Shin J Chang N. Phosphorylation of EZH2 activates STAT3 signaling *via* STAT3 methylation and promotes tumorigenicity of glioblastoma stem-like cells. Cancer Cell (2013) 23(6):839–52. doi: 10.1016/j.ccr.2013.04.008 PMC4109796 23684459

[68] Hsieh YY Lo HL Yang PM. EZH2 inhibitors transcriptionally upregulate cytotoxic autophagy and cytoprotective unfolded protein response in human colorectal cancer cells. Am J Cancer Res (2016) 6(8):1661–80. PMC5004071 27648357

[69] Qian C Yang C Tang Y Zheng W Zhou Y Song M. Pharmacological manipulation of Ezh2 with salvianolic acid B results in tumor vascular normalization and synergizes with cisplatin and T cell-mediated immunotherapy. Pharmacol Res (2022) 182:106333. doi: 10.1016/j.phrs.2022.106333 35779815

[70] Sun Y Jin L Liu JH Sui YX Han LL Shen XL. Interfering EZH2 expression reverses the cisplatin resistance in human ovarian cancer by inhibiting autophagy. Cancer Biother Radiopharm. (2016) 31(7):246–52. doi: 10.1089/cbr.2016.2034 27610467

[71] Jin Q Yu LR Wang L Zhang Z Kasper LH Lee JE. Distinct roles of GCN5/PCAF-mediated H3K9ac and CBP/p300-mediated H3K18/27ac in nuclear receptor transactivation. EMBO J (2011) 30(2):249–62. doi: 10.1038/emboj.2010.318 PMC3025463 21131905

[72] Park SE Yi HJ Suh N Park YY Koh JY Jeong SY. Inhibition of EHMT2/G9a epigenetically increases the transcription of beclin-1 *via* an increase in ROS and activation of NF-kappaB. Oncotarget (2016) 7(26):39796–808. doi: 10.18632/oncotarget.9290 PMC5129971 27174920

[73] Artal-Martinez de Narvajas A Gomez TS Zhang JS Mann AO Taoda Y Gorman JA. Epigenetic regulation of autophagy by the methyltransferase G9a. Mol Cell Biol (2013) 33(20):3983–93. doi: 10.1128/MCB.00813-13 PMC3811684 23918802

[74] Greer EL Maures TJ Hauswirth AG Green EM Leeman DS Maro GS. Members of the H3K4 trimethylation complex regulate lifespan in a germline-dependent manner in c. elegans. Nature. (2010) 466(7304):383–7. doi: 10.1038/nature09195 PMC3075006 20555324

[75] Fang R Barbera AJ Xu Y Rutenberg M Leonor T Bi Q. Human LSD2/KDM1b/AOF1 regulates gene transcription by modulating intragenic H3K4me2 methylation. Mol Cell (2010) 39(2):222–33. doi: 10.1016/j.molcel.2010.07.008 PMC3518444 20670891

[76] Gonzalez-Jaramillo V Portilla-Fernandez E Glisic M Voortman T Bramer W Chowdhury R. The role of DNA methylation and histone modifications in blood pressure: a systematic review. J Hum Hypertens (2019) 33(10):703–15. doi: 10.1038/s41371-019-0218-7 31346255

[77] Shin HJ Kim H Oh S Lee JG Kee M Ko HJ. AMPK-SKP2-CARM1 signalling cascade in transcriptional regulation of autophagy. Nature (2016) 534(7608):553–7. doi: 10.1038/nature18014 PMC5568428 27309807

[78] Suresh S Huard S Dubois T. CARM1/PRMT4: making its mark beyond its function as a transcriptional coactivator. Trends Cell Biol (2021) 31(5):402–17. doi: 10.1016/j.tcb.2020.12.010 33485722

[79] Kullo IJ Ding K Boerwinkle E Turner ST Mosley TH Jr. Kardia SL. Novel genomic loci influencing plasma homocysteine levels. Stroke (2006) 37(7):1703–9. doi: 10.1161/01.STR.0000225929.96190.b3 16741189

[80] Song H Feng X Zhang M Jin X Xu X Wang L. Crosstalk between lysine methylation and phosphorylation of ATG16L1 dictates the apoptosis of hypoxia/reoxygenation-induced cardiomyocytes. Autophagy (2018) 14(5):825–44. doi: 10.1080/15548627.2017.1389357 PMC6070011 29634390

[81] Yin Z Chen C Yang J Feng W Liu X Zuo R. Histone acetyltransferase MoHat1 acetylates autophagy-related proteins MoAtg3 and MoAtg9 to orchestrate functional appressorium formation and pathogenicity in magnaporthe oryzae. Autophagy (2019) 15(7):1234–57. doi: 10.1080/15548627.2019.1580104 PMC6613890 30776962

[82] Kong J Kong F Gao J Zhang Q Dong S Gu F. YC-1 enhances the anti-tumor activity of sorafenib through inhibition of signal transducer and activator of transcription 3 (STAT3) in hepatocellular carcinoma. Mol Cancer. (2014) 13:7. doi: 10.1186/1476-4598-13-7 24418169 PMC3895679

[83] Mazure NM Pouyssegur J. Hypoxia-induced autophagy: cell death or cell survival? Curr Opin Cell Biol (2010) 22(2):177–80. doi: 10.1016/j.ceb.2009.11.015 20022734

[84] Duga B Czako M Komlosi K Hadzsiev K Torok K Sumegi K. Deletion of 4q28.3-31.23 in the background of multiple malformations with pulmonary hypertension. Mol Cytogenet (2014) 7:36. doi: 10.1186/1755-8166-7-36 24959202 PMC4066825

[85] Cheng Z Du Z Zhai B Yang Z Zhang T. U1 small nuclear RNA overexpression implicates autophagic-lysosomal system associated with AD. Neurosci Res (2018) 136:48–55. doi: 10.1016/j.neures.2018.01.006 29395359

[86] Luo HR Moreau GA Levin N Moore MJ. The human Prp8 protein is a component of both U2- and U12-dependent spliceosomes. RNA (1999) 5(7):893–908. doi: 10.1017/s1355838299990520 10411133 PMC1369814

[87] Xu G Li T Chen J Li C Zhao H Yao C. Autosomal dominant retinitis pigmentosa-associated gene PRPF8 is essential for hypoxia-induced mitophagy through regulating ULK1 mRNA splicing. Autophagy (2018) 14(10):1818–30. doi: 10.1080/15548627.2018.1501251 PMC6135625 30103670

[88] Yoshida K Sanada M Shiraishi Y Nowak D Nagata Y Yamamoto R. Frequent pathway mutations of splicing machinery in myelodysplasia. Nature (2011) 478(7367):64–9. doi: 10.1038/nature10496 21909114

[89] Lv Y Zhang W Zhao J Sun B Qi Y Ji H. SRSF1 inhibits autophagy through regulating bcl-x splicing and interacting with PIK3C3 in lung cancer. Signal Transduct Target Ther (2021) 6(1):108. doi: 10.1038/s41392-021-00495-6 33664238 PMC7933324

[90] Ji E Lee H Ahn S Jung M Lee SH Lee JH. Heterogeneous nuclear ribonucleoprotein A1 promotes the expression of autophagy-related protein 6 in human colorectal cancer. Biochem Biophys Res Commun (2019) 513(1):255–60. doi: 10.1016/j.bbrc.2019.03.179 30954215

[91] Woodcock CC Hafeez N Handen A Tang Y Harvey LD Estephan LE. Matrix stiffening induces a pathogenic QKI-miR-7-SRSF1 signaling axis in pulmonary arterial endothelial cells. Am J Physiol Lung Cell Mol Physiol (2021) 320(5):L726–38. doi: 10.1152/ajplung.00407.2020 PMC8174827 33565360

[92] Wei R Chen L Li P Lin C Zeng Q. IL-13 alleviates idiopathic pulmonary hypertension by inhibiting the proliferation of pulmonary artery smooth muscle cells and regulating macrophage infiltration. Am J Transl Res (2022) 14(7):4573–90. PMC9360879 35958460

[93] Tang Y Zha L Zeng X Yu Z. Identification of biomarkers related to systemic sclerosis with or without pulmonary hypertension using Co-expression analysis. J Comput Biol (2020) 27(10):1519–31. doi: 10.1089/cmb.2019.0492 32298610

[94] Isakson P Holland P Simonsen A. The role of ALFY in selective autophagy. Cell Death Differ (2013) 20(1):12–20. doi: 10.1038/cdd.2012.66 22653340 PMC3524637

[95] Tranchevent LC Aube F Dulaurier L Benoit-Pilven C Rey A Poret A. Identification of protein features encoded by alternative exons using exon ontology. Genome Res (2017) 27(6):1087–97. doi: 10.1101/gr.212696.116 PMC5453322 28420690

[96] Bargiela A Sabater-Arcis M Espinosa-Espinosa J Zulaica M Lopez de Munain A Artero R. Increased muscleblind levels by chloroquine treatment improve myotonic dystrophy type 1 phenotypes in *in vitro* and *in vivo* models. Proc Natl Acad Sci U S A. (2019) 116(50):25203–13. doi: 10.1073/pnas.1820297116 PMC6911202 31754023

[97] Cogan J Austin E Hedges L Womack B West J Loyd J. Role of BMPR2 alternative splicing in heritable pulmonary arterial hypertension penetrance. Circulation (2012) 126(15):1907–16. doi: 10.1161/CIRCULATIONAHA.112.106245 PMC4113011 22923426

[98] Niu YN Liu QQ Zhang SP Yuan N Cao Y Cai JY. Alternative messenger RNA splicing of autophagic gene beclin 1 in human b-cell acute lymphoblastic leukemia cells. Asian Pac J Cancer Prev (2014) 15(5):2153–8. doi: 10.7314/apjcp.2014.15.5.2153 24716949

[99] Cheng B Xu A Qiao M Wu Q Wang W Mei Y. BECN1s, a short splice variant of BECN1, functions in mitophagy. Autophagy (2015) 11(11):2048–56. doi: 10.1080/15548627.2015.1100785 PMC4824595 26649941

[100] Jin Y Choi AM. Cross talk between autophagy and apoptosis in pulmonary hypertension. Pulm Circ (2012) 2(4):407–14. doi: 10.4103/2045-8932.105029 PMC3555411 23372925

[101] Ivanovska J Shah S Wong MJ Kantores C Jain A Post M. mTOR-Notch3 signaling mediates pulmonary hypertension in hypoxia-exposed neonatal rats independent of changes in autophagy. Pediatr Pulmonol. (2017) 52(11):1443–54. doi: 10.1002/ppul.23777 28759157

[102] Zhang MQ Li JR Peng ZG Zhang JP. Differential effects of autophagy-related 10 protein on HCV replication and autophagy flux are mediated by its Cysteine(44) and Cysteine(135). Front Immunol (2018) 9:2176. doi: 10.3389/fimmu.2018.02176 30319633 PMC6165859

[103] Sun Q Fan W Chen K Ding X Chen S Zhong Q. Identification of barkor as a mammalian autophagy-specific factor for beclin 1 and class III phosphatidylinositol 3-kinase. Proc Natl Acad Sci U S A. (2008) 105(49):19211–6. doi: 10.1073/pnas.0810452105 PMC2592986 19050071

[104] Diao J Liu R Rong Y Zhao M Zhang J Lai Y. ATG14 promotes membrane tethering and fusion of autophagosomes to endolysosomes. Nature (2015) 520(7548):563–6. doi: 10.1038/nature14147 PMC4442024 25686604

[105] Greer SU Chen J Ogmundsdottir MH Ayala C Lau BT Delacruz RGC. Germline variants of ATG7 in familial cholangiocarcinoma alter autophagy and p62. Sci Rep (2022) 12(1):10333. doi: 10.1038/s41598-022-13569-4 35725745 PMC9209431

[106] Gammoh N. The multifaceted functions of ATG16L1 in autophagy and related processes. J Cell Sci (2020) 133(20):jcs249227. doi: 10.1242/jcs.249227 33127840

[107] Lystad AH Carlsson SR de la Ballina LR Kauffman KJ Nag S Yoshimori T. Distinct functions of ATG16L1 isoforms in membrane binding and LC3B lipidation in autophagy-related processes. Nat Cell Biol (2019) 21(3):372–83. doi: 10.1038/s41556-019-0274-9 PMC7032593 30778222

[108] Fujiwara Y Furuta A Kikuchi H Aizawa S Hatanaka Y Konya C. Discovery of a novel type of autophagy targeting RNA. Autophagy (2013) 9(3):403–9. doi: 10.4161/auto.23002 PMC3590259 23291500

[109] Bryant A Palma CA Jayaswal V Yang YW Lutherborrow M Ma DD. miR-10a is aberrantly overexpressed in Nucleophosmin1 mutated acute myeloid leukaemia and its suppression induces cell death. Mol Cancer. (2012) 11:8. doi: 10.1186/1476-4598-11-8 22348345 PMC3306826

[110] Wu H Wang F Hu S Yin C Li X Zhao S. MiR-20a and miR-106b negatively regulate autophagy induced by leucine deprivation *via* suppression of ULK1 expression in C2C12 myoblasts. Cell Signal (2012) 24(11):2179–86. doi: 10.1016/j.cellsig.2012.07.001 22781751

[111] Huang Y Chuang AY Ratovitski EA. Phospho-DeltaNp63alpha/miR-885-3p axis in tumor cell life and cell death upon cisplatin exposure. Cell Cycle (2011) 10(22):3938–47. doi: 10.4161/cc.10.22.18107 PMC3266119 22071691

[112] Yang J Chen D He Y Melendez A Feng Z Hong Q. MiR-34 modulates caenorhabditis elegans lifespan *via* repressing the autophagy gene atg9. Age (Dordr). (2013) 35(1):11–22. doi: 10.1007/s11357-011-9324-3 22081425 PMC3543738

[113] Shi G Shi J Liu K Liu N Wang Y Fu Z. Increased miR-195 aggravates neuropathic pain by inhibiting autophagy following peripheral nerve injury. Glia (2013) 61(4):504–12. doi: 10.1002/glia.22451 23361941

[114] Huang Y Guerrero-Preston R Ratovitski EA. Phospho-DeltaNp63alpha-dependent regulation of autophagic signaling through transcription and micro-RNA modulation. Cell Cycle (2012) 11(6):1247–59. doi: 10.4161/cc.11.6.19670 PMC3335921 22356768

[115] Korkmaz G le Sage C Tekirdag KA Agami R Gozuacik D. miR-376b controls starvation and mTOR inhibition-related autophagy by targeting ATG4C and BECN1. Autophagy (2012) 8(2):165–76. doi: 10.4161/auto.8.2.18351 22248718

[116] Yu Y Yang L Zhao M Zhu S Kang R Vernon P. Targeting microRNA-30a-mediated autophagy enhances imatinib activity against human chronic myeloid leukemia cells. Leukemia (2012) 26(8):1752–60. doi: 10.1038/leu.2012.65 22395361

[117] Comincini S Allavena G Palumbo S Morini M Durando F Angeletti F. microRNA-17 regulates the expression of ATG7 and modulates the autophagy process, improving the sensitivity to temozolomide and low-dose ionizing radiation treatments in human glioblastoma cells. Cancer Biol Ther (2013) 14(7):574–86. doi: 10.4161/cbt.24597 PMC3742487 23792642

[118] Atala A. Re: VHL-regulated miR-204 suppresses tumor growth through inhibition of LC3B-mediated autophagy in renal clear cell carcinoma. J Urol. (2012) 188(6):2434. doi: 10.1016/j.juro.2012.08.067 23141280

[119] Meenhuis A van Veelen PA de Looper H van Boxtel N van den Berge IJ Sun SM. MiR-17/20/93/106 promote hematopoietic cell expansion by targeting sequestosome 1-regulated pathways in mice. Blood (2011) 118(4):916–25. doi: 10.1182/blood-2011-02-336487 PMC3148171 21628417

[120] Jing X Wu S Liu Y Wang H Huang Q. Circular RNA Sirtuin1 represses pulmonary artery smooth muscle cell proliferation, migration and autophagy to ameliorate pulmonary hypertension *via* targeting microRNA-145-5p/protein kinase-B3 axis. Bioengineered (2022) 13(4):8759–71. doi: 10.1080/21655979.2022.2036302 PMC9161928 35369850

[121] Zhang J Li Y Chen Y Yu X Wang S Sun H. Circ-calm4 regulates hypoxia-induced pulmonary artery smooth muscle autophagy by binding purb. J Mol Cell Cardiol (2023) 176:41–54. doi: 10.1016/j.yjmcc.2023.01.009 36716953

[122] Liu T Zou XZ Huang N Ge XY Yao MZ Liu H. Down-regulation of miR-204 attenuates endothelial-mesenchymal transition by enhancing autophagy in hypoxia-induced pulmonary hypertension. Eur J Pharmacol (2019) 863:172673. doi: 10.1016/j.ejphar.2019.172673 31542480

[123] Travers JG Wennersten SA Pena B Bagchi RA Smith HE Hirsch RA. And blocks covert extracellular matrix remodeling. Circulation (2021) 143(19):1874–90. doi: 10.1161/CIRCULATIONAHA.120.046462 PMC8884170 33682427

[124] Wang J Saren G Jiang H. HDAC inhibition: a novel therapeutic target for attenuating pulmonary hypertension by regulating tregs. Int J Cardiol (2015) 198:176–7. doi: 10.1016/j.ijcard.2015.06.172 26163911

[125] Humbert M McLaughlin V Gibbs JSR Gomberg-Maitland M Hoeper MM Preston IR. Sotatercept for the treatment of pulmonary arterial hypertension. N Engl J Med (2021) 384(13):1204–15. doi: 10.1056/NEJMoa2024277 33789009

[126] Thenappan T Ormiston ML Ryan JJ Archer SL. Pulmonary arterial hypertension: pathogenesis and clinical management. BMJ (2018) 360:j5492. doi: 10.1136/bmj.j5492 29540357 PMC6889979

[127] Wu YC Wang WT Lee SS Kuo YR Wang YC Yen SJ. Glucagon-like peptide-1 receptor agonist attenuates autophagy to ameliorate pulmonary arterial hypertension through Drp1/NOX- and atg-5/Atg-7/Beclin-1/LC3beta pathways. Int J Mol Sci (2019) 20(14):3435. doi: 10.3390/ijms20143435 31336911 PMC6678531

[128] Zhou Y Wang Y Wang X Tian X Zhang S Yang F. The protective effects of kappa-opioid receptor stimulation in hypoxic pulmonary hypertension involve inhibition of autophagy through the AMPK-MTOR pathway. Cell Physiol Biochem (2017) 44(5):1965–79. doi: 10.1159/000485886 29224002

[129] Wu K Zhang Q Wu X Lu W Tang H Liang Z. Chloroquine is a potent pulmonary vasodilator that attenuates hypoxia-induced pulmonary hypertension. Br J Pharmacol (2017) 174(22):4155–72. doi: 10.1111/bph.13990 PMC5659991 28849593

